# Refractory Spontaneous Bacterial Empyema in Cirrhotic Patient

**DOI:** 10.1155/2021/6685998

**Published:** 2021-07-14

**Authors:** Erica Chow, Bashar Khiatah, Amanda Frugoli

**Affiliations:** ^1^Western University of Health Sciences, Pomona, Community Memorial Hospital, 147 N Brent St, Ventura, CA 93003, USA; ^2^Department of Internal Medicine, Community Memorial Hospital, 147 N Brent St, Ventura, CA 93003, USA; ^3^Department of Graduate Medical Education, Department of Internal Medicine, Community Memorial Hospital, 147 N Brent St, Ventura, CA 93003, USA

## Abstract

Spontaneous bacterial empyema (SBEM), also called spontaneous bacterial pleuritis, is an infection of the pleural space that arises in the setting of cirrhosis and, by definition, the absence of pneumonia. It is likely underdiagnosed as its symptoms are nonspecific and it lacks standardized diagnostic and therapeutic recommendations. SBEM represents a distinct complication of hepatic hydrothorax with different pathogenesis, presentation, and treatment strategy from those of empyema secondary to pneumonia. Surprisingly, nearly 40% of episodes of spontaneous empyema are not associated with spontaneous bacterial peritonitis. Although SBEM is amenable to prompt antibiotic therapy, it has a high rate of mortality and morbidity. A high clinical suspicion is crucial for patient survival and timely initiation of appropriate antibiotics. Increased understanding, recognition, and standardization of treatment would help alleviate the relatively high burden of SBEM. In this case vignette, we provide a review of the relevant literature, and we describe a rare case of SBEM in a patient with a history of alcohol-associated liver cirrhosis and prior episode of spontaneous bacterial peritonitis (SBP). SBEM was diagnosed with thoracentesis and analysis of the aspirate, and he was treated with ceftriaxone with resolution of his presenting abdominal pain and leukocytosis.

## 1. Introduction

Spontaneous bacterial empyema (SBEM) is a rare infection of an existing hepatic hydrothorax in the absence of underlying pneumonia. This is the primary distinguishing feature from the more common parapneumonic empyema. By definition, the fluid of empyema in both SBEM and parapneumonic is purulent, containing many inflammatory cells and sometimes culture positive. In contradiction, the fluid of parapneumonic effusions and hepatic hydrothorax is sterile with few to no inflammatory cells present. Although it was first described by Flaum in 1976, it remains an elusive and likely underdiagnosed pathology. Understanding SBEM is essential in timely diagnosis by thoracentesis and initiation of medical therapy for these patients. It is crucial to recognize and distinguish between these disease processes as SBEM represents a distinct complication of hepatic hydrothorax with different pathogenesis, presentation, and treatment strategy from those of empyema secondary to pneumonia. In this case vignette, we describe a patient with a known alcoholic liver cirrhosis who presented with abdominal pain and was determined to have SBEM and review relevant information regarding this disease.

## 2. Clinical Case

A 65-year-old male with a long history of alcohol abuse and cirrhosis presented to the emergency department for abdominal pain described as constant and nagging pain for the last couple of weeks. He had difficulty locating the source of his pain but pointed to his umbilical hernia which had been diagnosed earlier that week.

His past medical history was notable for alcohol dependence, cirrhosis with a Model for End-Stage Liver Disease (MELD) score of 27, chronic pain disorder with opioid dependence, gastroesophageal reflux disease, and hypertension. He also had a lengthy history of multiple emergency department visits and admissions in the preceding years related to alcohol abuse and decompensated cirrhosis. A few months before this admission, he presented with abdominal pain and ascites and was found to have *E. coli* bacteremia from spontaneous bacterial peritonitis (SBP). This was successfully treated with ceftriaxone and metronidazole and discharge home on oral antibiotics for 14 days. For the last 40 years, he drank about a pint of vodka daily, although he reported abstinence since his SBP admission a few months ago. He has a history of marijuana use and a 40-year exposure to second-hand tobacco smoke.

In the Emergency Department (ED), he was initially afebrile with a pulse of 91 beats per minute, a blood pressure of 108/64, and 100% oxygen saturation on room air. On physical exam, he was visibly jaundiced and ill-appearing. His abdominal exam demonstrated a soft, 4 × 4 cm umbilical hernia which was minimally tender to palpation and easily reducible, as well as diffuse abdominal tenderness. The remainder of his exam was unremarkable, with normal cardiorespiratory, neurologic, and jaundiced skin findings. Initial lab tests are shown in Table 1.

An initial differential diagnosis included intra-abdominal infection, hepatobiliary disease, abdominal hernia, and bowel obstruction. Intra-abdominal infection was deemed likely with his diffuse abdominal pain resembling his prior admission for SBP, elevated white blood cells, and cirrhosis history. Paracentesis was initially attempted in the ED, but no adequate fluid pocket was visualized on ultrasound. Ascitic fluid remained minimal over the next few days, and paracentesis was ultimately deferred in favor of empiric treatment for SBP. The intrabiliary disease was also a concern due to his elevated total bilirubin and alkaline phosphatase levels.

**Figure 1:**
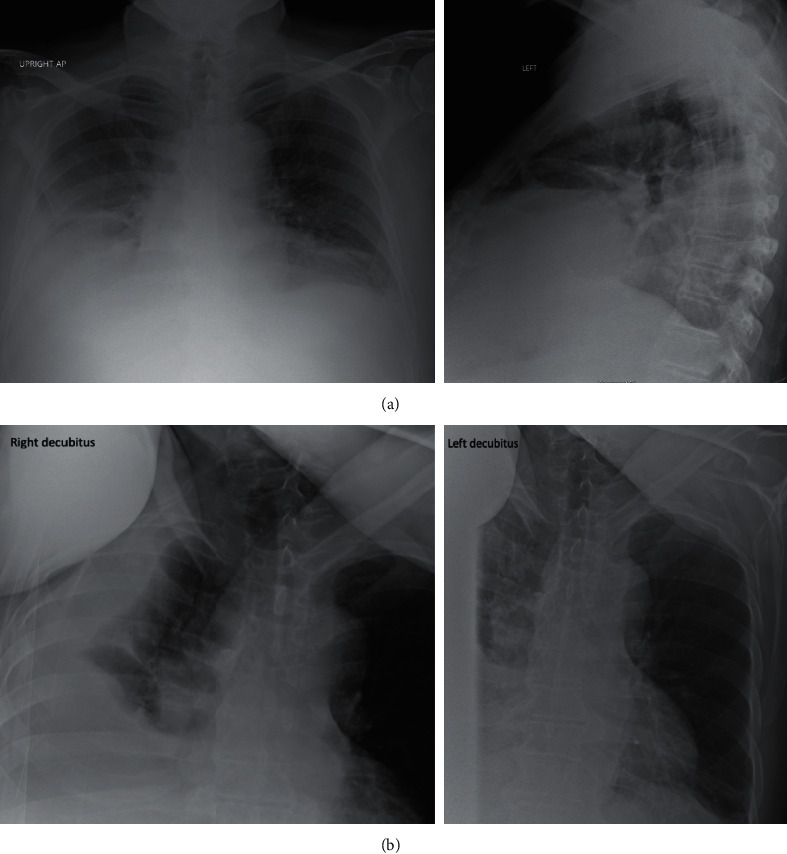
(a) Portable upright chest radiography showing blunting of the right costophrenic angle consistent with small-to-moderate right pleural effusion and linear meniscus with compression atelectasis. (b) Chest X-ray in right decubitus position, showing a large, layering pleural effusion on the right with a possible location at the right lung base.

**Figure 2:**
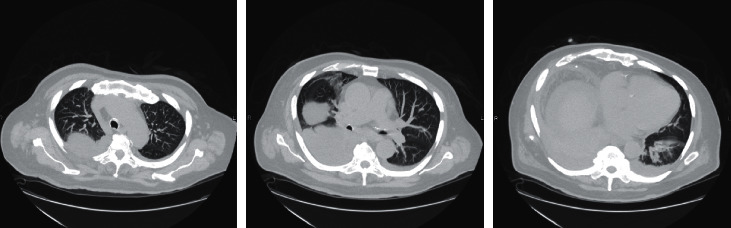
Computed tomography of the chest, showing a large, loculated effusion on the right suspicious for empyema.

The patient met the criteria for severe inflammatory response syndrome with leukocytosis and tachycardia and was admitted for sepsis. Computed tomography of the abdomen confirmed a ventral abdominal hernia, cirrhosis, hepatosplenomegaly without hepatic mass, and a small right pleural effusion. Since the patient described his symptoms as similar to his previous admission, recurrent SBP was suspected and empiric ceftriaxone was started. Given his sepsis parameters, empiric metronidazole was initiated for anaerobic coverage until his source could be confirmed. Ultrasound-guided paracentesis was attempted, but due to small-volume ascites and no adequate fluid pocket, the procedure was aborted. His epigastric pain elevated alkaline phosphatase, and hyperbilirubinemia warranted a magnetic resonance cholangiopancreatography which identified a mass in the pancreas. This was subsequently biopsied during this hospitalization and confirmed pancreatic adenocarcinoma. His sepsis parameters improved with downtrending of his white blood cell count over the next couple of days; however, a sharp increase in leukocytosis was noted on hospital day 3 (26.3, compared to 18.1 on admission). This worsening leukocytosis was accompanied by fevers, chills, and increased oxygen requirements, raising concern for pneumonia. A chest X-ray series including a decubitus view showed a large right pleural effusion (Figures 1(a) and 1(b)). A computed tomography chest scan then confirmed a large, loculated right-sided pleural effusion and severe compression atelectasis (Figure 2). Empyema was suspected, and a thoracentesis was performed. 1400 ml of dark amber fluid was removed without complication. Analysis of the thoracentesis fluid is reported in Table 2. Based on the thoracentesis aspirate white blood cell count of 1,622 cells/mm^3^ and the absence of pneumonia, the patient was confirmed to have SBEM.

## 3. Management

At the time of his admission, recurrent SBP was suspected and empiric ceftriaxone and metronidazole were initiated. When results of his thoracentesis indicated SBEM, the antimicrobials were escalated to intravenous vancomycin, cefepime, and metronidazole. Infectious disease service was consulted and recommended continuation of these during hospitalization. The patient required a lengthy inpatient stay for a total of 26 days. He was discharged with a prescription for two more weeks of oral antibiotics and follow-up with infectious disease to discuss antibiotic prophylaxis.

## 4. Outcome and Follow-Up

In regards to his SBEM, the patient showed significant clinical improvement within the first few days of thoracentesis and adjusted antibiotic therapy. His leukocytosis resolved, and subsequent chest X-ray showed resolution of the empyema. He elected to treat the pancreatic adenocarcinoma with chemotherapy and was ultimately discharged in a stable condition.

## 5. Discussion

The pathophysiology of SBEM is still unclear and could possibly have two separate etiologies. Cirrhosis is a unifying factor by definition; the incidence of SBEM in cirrhotic patients with hydrothorax is 13–16%, and 2–2.4% in cirrhotic patients without hydrothorax [1]. Risk factors for developing SBEM include a high Child-Pugh score or preexisting SBP [2–4]. It is postulated that SBP could expand from the perihepatic region to involve the pleural space [5]. It is possible that the hydrostatic pressure from ascites allows the movement of ascites fluid and bacteria present through fenestrations in the diaphragm into the pleural space. This could also occur in the absence of large-volume abdominal ascites as ascites fluid can follow the pressure gradient from a high abdominal cavity pressure to the lower pressure of the pleural space. However, 44% of SBEM cases arise in the absence of SBP [1]. Even though SBP and SBEM share similarities and may share a similar pathogenesis, they are separate entities [4, 6]. Alternatively, it is hypothesized that preexisting hepatic hydrothorax compounded with bacteremia can lead to infection of the pleural space and lead to SBEM [4, 7]. In this model of hematogenous seeding, microorganisms reach the pleural space via bacteremia and infect preexisting hepatic hydrothorax, creating an empyema [8]. However, this model does not account for the nearly 70% of SBEM cases that arise in the absence of bacteremia [4, 6]. A compounding factor for both includes reduced immune response for patients with cirrhosis. As cirrhosis results in low complement level and decreased opsonic activity that can lead to reduced reticuloendothelial phagocyte activity [2, 8]. Other documented risk factors for the development of SBEM include low serum albumin, low pleural fluid protein, and decreased C3 levels [2–4].

SBEM diagnostic criteria are met with either a positive pleural fluid culture with a pleural fluid polymorphonuclear cell count >250 cells/mm^3^ or a negative culture with a pleural fluid neutrophil count >500 cells/mm^3^ without evidence of pneumonia on chest imaging [9]. Signs and symptoms of SBEM are nonspecific; thus, a high index of suspicion is needed in order to identify these patients [7, 10]. A cirrhotic patient who experiences clinical deterioration or a leukocytosis spike without a clear etiology should undergo diagnostic thoracentesis [8]. The inclusion of “empyema” in SBEM nomenclature can be misleading, so it should be noted that pleural fluid can be classified as transudate rather than exudate in up to half of SBEM cases [1]. It should also be noted that Light's criteria have limited efficacy in application with hepatic hydrothorax and SBEM, as up to 18% of hepatic hydrothorax cases are misclassified as exudative [11]. Workup should begin with chest imaging to rule out pneumonia [9]. Thoracentesis is the next step in establishing the diagnosis of SBEM and should include cultures and analysis of the pleural fluid for a definitive diagnosis [9]. Additionally, leukocyte esterase urine reagent test strips can be repurposed as a rapid and inexpensive indicator of SBEM from thoracentesis fluid, expediting the initiation of empiric antibiotics [12].

Mortality rates for SBEM patients can be as high as 20–38%; thus, prompt and appropriately aggressive management of SBEM and underlying liver disease is critical to patient survival [2, 6, 10]. SBEM should be treated similarly to SBP [13]. Common culprits in SBEM are *Escherichia coli*, followed by *Streptococcus* species, *Enterococcus*, *Klebsiella*, or *Pseudomonas* [1], which are parallel to those seen in SBP [1, 9]. First-line antibiotic therapy is a third-generation cephalosporin, which should be initiated early based on high clinical suspicion for SBEM [1, 8, 10]. While the standard of care for parapneumonic empyema involves the placement of a chest tube for drainage and can require video-assisted thoracoscopic surgery with debridement and talc pleurodesis, this is not the same for SBEM. The appropriate SBEM management includes IV antibiotics and the management of hydrothorax [1, 14, 15]. Hepatic hydrothorax should be managed closely with a low-sodium diet and diuretics including spironolactone and a loop diuretic. For patients with refractory disease that are not transplant candidates, treatment with transjugular intrahepatic portosystemic shunt placement could be considered as clinically indicated [16]. It should be noted that talc pleurodesis with surgical videothoracoscopy with closure of diaphragmatic defects has been attempted for this condition but with poor outcomes. [14, 17] Once the patient has shown clinical improvement, prophylactic oral antibiotics should be considered to mitigate the high risk of recurrence [18]. For cases with simultaneous SBP, clinicians should follow the latest AASLD guidelines. Current recommendations include long-term prophylaxis with daily norfloxacin or trimethoprim/sulfamethoxazole for patients who have survived an episode of SBP. This recommendation is class I and level A. It should be noted that the occurrence of SBEM should not impact consideration for, or patient's standing on the liver transplant list, as patients with SBEM do as well as patients with uncomplicated hepatic hydrothorax [1, 7].

## 6. Conclusion

SBEM is a relatively uncommon occurrence in patients with cirrhosis and hydrothorax, and it remains an underdiagnosed pathology. The under-recognition of SBEM can be attributed in part to its poorly understood pathogenesis, nonspecific symptom presentation, and hesitation or delay in diagnostic thoracentesis. Further elucidation of the SBEM pathogenesis and prevalence could improve our understanding and clinical recognition of this entity. Increased awareness of the presentation of SBEM can expedite the time to diagnosis and initiation of prompt and appropriate antibiotic therapy. Finally, standardization of diagnostic and therapeutic guidelines would help to alleviate the relatively high morbidity and mortality burden associated with SBEM. Diagnostic criteria, diagnostic steps, and treatment recommendations are summarized in Table 3.

## Figures and Tables

**Table 1:** Laboratory results.

		Normal range
White blood cell count	23.8 k/uL	4.8–10.8 k/uL
Red blood cell count	3.97 m/uL	4.7–6.10 m/uL
Hemoglobin	12.7 g/dL	12.0–18.0 g/dL
Hematocrit	38.5%	42.0–52.0%
Mean corpuscular volume	97.0 fL	80–94 fL
Platelet estimate	Normal	130–400 k/uL
Total bilirubin	14.9 mg/dL	0.1–1.3 mg/dL
Aspartate aminotransferase	38 mg/dL	12–45 IU/L
Alanine aminotransferase	34 mg/dL	2–40 IU/L
Alkaline phosphatase	161 IU/L	41–133 IU/L
Total protein	6.7 g/dL	6.0–8.0 g/dL
Albumin	1.8 g/dL	3.5–4.8 g/dL
Lipase	61 U/L	22–50 IU/L

**Table 2:** Thoracentesis results.

White Blood Cell Count	1622 k/uL
Red blood cell count	20889 m/uL
Neutrophil count	49 k/uL
Lymphocytes	51 k/uL
Gram stain	No growth
Lactate dehydrogenase	232 U/L
Amylase	14 U/L
Albumin	1.2 g/dL
Total protein	3.3 g/dL
Glucose	204 mg/dL
pH	7.67

**Table 3:** Summary of recommendations.

Diagnostic criteria	1. No evidence of pneumonia on chest imaging.	
	2. Positive pleural fluid culture and PMN cell count >250 cells/mm^3^.	or	Negative pleural fluid culture and PMN cell count >500 cells/mm^3^.

Diagnostic workup	Chest imaging to rule out pneumonia. Diagnostic thoracentesis.		

Treatment	First-line antibiotic therapy: third-generation cephalosporin. Manage hepatic hydrothorax and underlying cirrhosis. Outpatient oral antibiotics to prevent a recurrence.		

PMN = polymorphonuclear.
